# *LTA4H* Genotype Is Associated with Susceptibility to Bacterial Meningitis but Is Not a Critical Determinant of Outcome

**DOI:** 10.1371/journal.pone.0118789

**Published:** 2015-03-23

**Authors:** Sarah J. Dunstan, Trinh Thi Bich Tram, Guy E. Thwaites, Tran Thi Hong Chau, Nguyen Hoan Phu, Tran Tinh Hien, Jeremy J. Farrar, Marcel Wolbers, Nguyen Thi Hoang Mai

**Affiliations:** 1 Oxford University Clinical Research Unit, Ho Chi Minh City, District 5, Vietnam; 2 Centre for Tropical Medicine, Nuffield Department of Clinical Medicine, Oxford University, Oxford, OX3 7LJ, United Kingdom; 3 Nossal Institute of Global Health, Melbourne School of Population and Global Health, University of Melbourne, Melbourne, 3010, Australia; 4 Hospital for Tropical Diseases, Ho Chi Minh City, District 5, Vietnam; University of New South Wales, AUSTRALIA

## Abstract

Adjunctive dexamethasone saves lives in the treatment of tuberculous meningitis but this response is influenced by the patient’s *LTA4H* genotype. Despite less certain benefit, adjunctive dexamethasone is also frequently used in the treatment of pyogenic bacterial meningitis, but the influence of *LTA4H* genotype on outcomes has not been previously investigated. We genotyped the *LTA4H* promoter region SNP (rs17525495) in 390 bacterial meningitis patients and 751 population controls. rs17525495 was associated with susceptibility to bacteriologically confirmed bacterial meningitis (P = 0.01, OR 1.27 95% confidence interval [CI] 1.05–1.54) but did not influence clinical presentation, disease severity or survival following dexamethasone treatment.

## Introduction

Vaccination against some of the causative agents of bacterial meningitis has reduced the burden of this disease in high-income countries [1]. Despite these advances in prevention, the burden of this life-threatening disease remains highest in resource-limited countries resulting in approximately 1.2 million cases of bacterial meningitis globally every year. To reduce the high mortality and morbidity of bacterial meningitis in developing countries, rapid diagnosis and initiation of effective antibiotics is crucial to avoid the devastating consequences of delayed treatment. Equally, strategies to control the inflammatory response in the subarachnoid space may also reduce mortality and decrease the rate of neurological sequelae that occurs in approximately 50% of survivors [2].

The treatment of bacterial meningitis has been the subject of a number of randomised controlled trials (RCT). The interventions investigated have included continuous antibiotic infusion, the optimal duration of antibiotic therapy, and adjunctive treatments such as glycerol and dexamethasone [3,4]. A RCT of adjunctive dexamethasone in Vietnam found dexamethasone was associated with increased survival, but only in patients with microbiologically confirmed bacterial meningitis [5]. A more recent meta-analysis of data from all published RCTs concluded that adjunctive corticosteroids reduces hearing loss and neurological sequelae following bacterial meningitis, but probably has no effect on mortality [4].

Leukotriene A4 hydrolase, encoded by *LTA4H*, catalyses the production of leukotriene B4 (LTB4), an eicosanoid that has potent chemoattractant and pro-inflammatory properties [6]. Recently we, and others reported that a SNP rs17525495 in the *LTA4H* promoter, regulating *LTA4H* transcription, determined inflammatory cell recruitment, patient survival and response to dexamethasone treatment in Vietnamese adults with tuberculous meningitis (TBM) [7]. Tobin *et al* [7,8] additionally showed that the balance of pro- and anti-inflammatory pathways (LTB4 and lipoxin A4, respectively), regulated by LTA4H, played a significant role in controlling bacterial growth and susceptibility to mycobacterial infections. In TBM patients receiving adjunctive dexamethasone, only individuals with the high expression *LTA4H* genotype, and therefore excess TNF and inflammation, benefitted from anti-inflammatory therapy [7,9]. This was one of the first examples where host genotype-specific therapies could influence survival from an infectious disease. Since the inflammatory network regulated by LTA4H may be fundamental to the host inflammatory response to diverse infections, we hypothesized that outcome of dexamethasone treatment in bacterial meningitis is dependent on *LTA4H* genotype.

## Methods

### Study subjects

Patients with suspected bacterial meningitis (N = 435) were recruited into a randomised controlled trial of adjunctive dexamethasone treatment in Ho Chi Minh City (HCMC), Vietnam [5]. Study patients were >14 years old, had clinical evidence of meningitis (headache, fever, vomiting and neck stiffness) and at least one of the following; bacteria in cerebrospinal fluid (CSF) detected by Gram’s or acridine orange stain; positive CSF fluid latex agglutination; pathogenic bacteria cultured from blood or CSF; or a clinical history of illness >7 days, with cloudy CSF, >60% neutrophils by white cell count, and CSF to blood glucose ratio <50%. Patients were randomly assigned to receive intravenous dexamethasone sodium phosphate, 0.4 mg per kg body weight, every 12 hours for 4 days, or placebo. The study medication was given 15 minutes before the administration of antibiotics, although some patients may have had prior antibiotic treatment. Upon discharge, patients were classified “definite bacterial meningitis” if bacteria were detected by CSF stain or CSF blood culture. “Probable meningitis” was defined as patients that were not microbiologically confirmed with no alternative diagnosis.

The population control individuals (N = 751) were cord blood control samples collected from babies born in 2003 at Hung Vuong Obstetric Hospital, HCMC, Vietnam, as described previously [10]

Verbal consent was obtained from all subjects with bacterial meningitis, or their parent/guardian/relative in the case of severe cases where the patient was unable to consider consent independently, i.e. unconscious. Obtaining verbal informed consent was confirmed by the assignment of a study identifier and completion of the study enrollment form. This consent procedure reflected current practise at the Hospital for Tropical Diseases and in Viet Nam during the study period (1996–2005). In Viet Nam, patients over 14 years of age are considered adults and treated within the adult care system. These patients have the autonomy to give independent consent for medical procedures, and for clinical research when approved by an Ethics Committee. This consent process, including recruitment of patients over 15 years of age to give independent consent to participate in this trial, was approved by the Scientific and Ethical Committee of the Hospital for Tropical Diseases, Ho Chi Minh City, in line with the standard health care system. The original clinical study describing these patients and detailing this approved consent process was published in 2007[5].

For cord blood control samples, written informed consent was obtained from the mother. Ethical approval for cord blood control collection was granted by the scientific and ethical committees at the Hospital for Tropical Diseases, Ho Chi Minh City and Hung Vuong Hospital, Ho Chi Minh City. The clinical study was also approved by the Oxford Tropical Research Ethics Committee, United Kingdom.

### DNA extraction and quantification

Genomic DNA from bacterial meningitis and cord blood controls was extracted from between 1–5ml of venous blood collected in tubes containing EDTA anti-coagulant. DNA was extracted by using the Nucleon BACC2 Genomic DNA extraction kits (GE Healthcare) or the blood maxi kit from Qiagen (Lewes, UK). DNA concentration was determined by Nanodrop.

### Genotyping

rs17525495 was genotyped by Taqman using a pre-designed assay kits (Applied Biosystems) according to the suppliers instructions (http://tools.lifetechnologies.com/content/sfs/manuals/TaqMan_SNP_Genotyping_Assays_man.pdf). This was performed using a LightCycler 480 Probes Master kit on the LightCycler 480 real-time PCR system (Roche) according to the suppliers protocol (https://pim-eservices.roche.com/LifeScience/Document/4dd0e207-97ed-e311-98a1-00215a9b0ba8).

### Clinical severity and inflammation indices

All clinical data were obtained from the previously published study [5]. CSF concentrations of TNF-α were measured in a sub-group of patients with microbiologically confirmed bacterial meningitis patients, as has been reported previously [11].

### Data Analysis

Genotypic deviations from Hardy-Weinberg equilibrium (HWE) were assessed using a chi-square statistical test. Univariate analysis was performed for categorical variables with Pearson’s *χ* ^2^ test to assess associations between disease phenotype and allele or genotype frequencies. Logistic regression of the case control association analysis allowed the odds ratios for the genotypes to be estimated. In this approach we modelled the SNP of interest assuming several related genotypic mechanisms (additive, recessive and general models). For more details of the general model, see Dunstan *et al* [10]. No corrections for multiple testing were performed.

When investigating severity and inflammation indices of bacterial meningitis patients by genotype, categorical variables were summarised by absolute count (%) and compared by the χ^2^ test, whereas continuous data were summarised by median (IQR) and compared by the Kruskall-Wallis test. One-month mortality among patients not treated and treated with dexamethasone adjunctive therapy, and stratified by rs17525495 genotype, were summarized with Kaplan-Meier curves. Comparisons between genotypes were based on Cox regression models adjusted for the randomized treatment group; the hypothesis that the effect of dexamethasone on mortality depends on the genotype was assessed with an interaction test. In addition to one-month mortality, we also analyzed the composite endpoint of death or severe neurological sequelae or bilateral severe deafness at 1 month [4] using logistic regression. All analyses were performed using the statistical package R v3.0.1 [12].

## Results and Discussion

### Clinical characteristics of cohort

435 Vietnamese adults with suspected bacterial meningitis were randomly allocated adjunctive dexamethasone or placebo in addition to standard antimicrobial therapy [5]. 423 were subsequently diagnosed as having either microbiologically confirmed bacterial meningitis (n = 300; 69%) or probable bacterial meningitis (n = 123; 28%) [5]. Only one of these patients was infected with HIV (419 tested). 12 adults (3%) had an alternative diagnosis and were excluded from the current study. In addition, DNA or genotype data was unavailable from 33 patients leaving 390 patients to be analysed in the genetics study. These patients were adults with a median (interquartile range [IQR]) age of 42 (29–55) years, of which 106 (27%) were female. Within the control group 49% of subjects were female. Additional molecular diagnostics implemented after the initial RCT resulted in 312 (80%) patients with a confirmed diagnosis of bacterial meningitis and the remaining 78 (20%) had “probable meningitis”. The most commonly identified bacterial agent in the patients with microbiologically confirmed bacterial meningitis was *Streptococcus suis* [123/312 (39%)] [5]. Other agents identified were *Streptococcus pneumoniae* [75/312 (24%)], *Neisseria meningitidis* [26/312 (8%)], *Haemophilus influenzae* [8/312 (3%)], aerobic Gram negative bacilli [18/312 (6%)] and others [62/312 (20%) which comprised a mix of *Streptococcus spp*., *Staphylococcus aureus*, coagulase-negative staphylococcus, *Klebsiella spp*., *Escherichia coli* and other gram negative bacteria] [5].

### The effect of *LTA4H* genotype on susceptibility to bacterial meningitis

We genotyped the *LTA4H* promoter SNP rs17525495, which has been previously associated with TBM, in 390 bacterial meningitis cases and 751 population controls. rs17525495 was in Hardy Weinberg Equilibrium in controls (P = 0.57) The frequency of the minor allele in Vietnamese controls was 0.33, whereas it is 0.04 in Caucasians (CEU), 0.29 in Chinese and Japanese (CHB+JPT) and 0.12 in Nigerians (YRI) (http://www.ncbi.nlm.nih.gov/SNP/). Table 1 shows that rs17525495 was associated with susceptibility to bacterial meningitis, both in patients with microbiologically confirmed bacterial meningitis and in those in whom the diagnosis was likely but unproven. In the additive model, rs17525495 was associated with bacteriologically confirmed bacterial meningitis (P = 0.01, OR 1.27, 95% confidence interval [CI] 1.05–1.54), with the recessive model of inheritance showing an increased risk of bacterial meningitis for TT homozygotes (P = 0.008, OR 1.65, 95% CI 1.15–2.39). rs17525495 was not significantly associated with BM under the heterozygote advantage model (P = 0.91, OR 0.99, 95% CI 0.77–1.26; data not shown).

**Table 1:** rs17525495 is associated with susceptibility to bacterial meningitis.

**subject**	**allele**	**genotype**	**freq**	***P***		***P***			***P***		**HWE ^*j*^**
**group**	**freq^*a*^ C^b^**	**CC/CT^c^/TT**	**CC/CT/TT**	**(add^*d*^)**	**OR (95% CI)^*e*^**	**(gen^*f*^)**	**OR (95% CI)^*g*^**	**OR (95% CI)^*h*^**	**(rec^*i*^)**	**OR (95% CI)**	**control *P***
control	0.67	339/326/86	0.45/0.43/0.11								0.57
BM all	0.61	155/168/67	0.40/0.43/0.17	0.01	1.26 (1.06–1.50)	0.02	1.13(0.86–1.47)	1.70 (1.18–2.47)	0.008	1.60 (1.13–2.27)	0.07
BM def^*k*^	0.61	124/133/55	0.40/0.43/0.18	0.01	1.27 (1.05–1.54)	0.02	1.12(0.84–1.49)	1.75 (1.18–2.60)	0.008	1.65 (1.15–2.39)	0.07

^*a*^frequency

^*b*^cytosine

^*c*^thymine

^*d*^additive model

^*e*^odds ratio (95% confidence interval)

^*f*^general model; a genotypic model where one genotype group (e.g. CC group) is baseline

^*g*^estimates of odds for general model, comparing baseline (CC) to CT

^*h*^estimates of odds for general model comparing baseline (CC) to TT

^*i*^recessive model

^*j*^Hardy Weinberg Equilibrium

^*k*^definite. NB: data for dominant and heterozygote advantage model were not significant and are not shown here.

Studies in zebra fish and humans with mycobacterial infection have suggested a human model of heterozygous advantage, with increased disease susceptibility experienced by those with either minor or major allele homozygosity [8]. Our current findings suggest only the TT homozygotes, with a presumed hyper-inflammatory phenotype, have increased susceptibility to bacterial meningitis. To date, mechanisms of host genetic susceptibility to the commonest causes of bacterial meningitis (*S*. *pneumoniae* and *N*. *meningitidis*) have generally evoked a failure of immune response. For example, there is a well-documented association between genetically determined deficiencies in the terminal complement components (C5–9) and meningococcal meningitis [13], and deficiencies in the soluble pattern recognition molecule mannose binding lectin have recently been associated with increased susceptibility to bacterial meningitis, especially pneumococcal meningitis [14]. Therefore, the association between an LTA4H hyper-inflammatory phenotype and the development of bacterial meningitis may represent a novel mechanism of susceptibility which needs further investigation in other pyogenic bacterial infections and confirmation in different populations.

### The effect of *LTA4H* genotype on presentation and response to treatment of bacterial meningitis

To determine if *LTA4H* genotype had any effect on the clinical parameters of bacterial meningitis we investigated baseline markers of clinical severity and the CSF inflammatory indices of the patients, stratified by rs17525495 genotype. *LTA4H* genotype was not associated with any significant difference in disease severity before the start of treatment, or difference in CSF inflammatory indices, regardless of whether the diagnosis was confirmed or probable (Table 2).

**Table 2:** Clinical severity and CSF inflammation indices of bacterial meningitis patients by rs17525495 genotype on study recruitment.

	**total**	**TT**	**TT Summary**	**CT**	**CT Summary**	**CC**	**CC Summary**	**Test**
**Characteristic**	**n**	**n**	**statistic^*a*^**	**n**	**statistic**	**n**	**statistic**	**statistic^*b*^**
**all BM**								
Glascow coma score	390	67	13 (10–15)	168	13 (9.75–15)	154	13 (9.35–15)	0.99
coma group^C^	390							
- normal		67	22 (33)	168	58 (35)	154	54 (35)	0.91
- mild impairment			23 (34)		62 (37)		49 (32)	
- moderate impairment			14 (21)		34 (20)		32 (21)	
- severe impairment			8 (12)		14 (8)		19 (12)	
opening pressure (cmHgCSF)	306	48	21 (14–43)	137	22 (16–50)	121	25 (14–46)	0.75
CSF white cell count / mm^3^	389	66	4505 (2025–9042.5)	168	2500 (1095–6788.5)	155	3280 (825–8600)	0.13
CSF Neutrophil percentage	386	66	90.5 (78.25–95)	166	85 (77–92)	154	90 (78–94)	0.07
CSF total number of Neutrophil / mm3	386	66	3733.3 (1571.5–7635)	166	2184.7 (922.2–5764.5)	154	2819.2 (666.9–7530.2)	0.19
Proportion of glucose in the CSF	387	67	17 (8.5–34.5)	166	25.5 (12–44.75)	154	27 (11–46)	0.13
Albumin in CSF (mg/dl)	382	66	265 (170–400)	163	239 (143.5–406)	153	228 (147–385)	0.47
**definite BM**								
Glascow coma score	312	55	13 (9–15)	133	13 (9–15)	124	13 (9–15)	0.8134
coma group	312							
- normal		55	16 (29)	133	44 (33)	124	40 (32)	0.80
- mild impairment			19 (35)		50 (38)		40 (32)	
- moderate impairment			12 (22)		29 (22)		29 (23)	
- severe impairment			8 (15)		10 (8)		15 (12)	
opening pressure (cmHgCSF)	245	38	25 (14.2–49.7)	109	22 (17–50)	98	25 (16.2–46)	0.99
CSF white cell count / mm^3^	311	54	4255 (1880–8952.5)	133	2620 (1100–6790)	124	3625 (847.5–9060.5)	0.31
CSF Neutrophil percentage	308	54	91 (79.25–97.75)	131	87 (78.5–93)	123	90 (78–95)	0.09
CSF total number of Neutrophil / mm3	308	54	3530.1 (1571.5–7635)	131	2352 (927–6054.5)	123	3465 (685.4–8087.8)	0.37
Proportion of glucose in the CSF	310	55	19 (9.5–34.5)	132	26.5 (10–46)	123	23 (9–46)	0.13
Albumin in CSF (mg/dl)	305	54	270 (170–396.5)	128	265 (148.5–428.5)	123	248 (160.5–430)	0.93
TNF-alpha in CSF (pg/ml)	126	23	812.95 (42.44–3550.55)	52	91.12 (30.28–897.32)	51	205.64 (37.68–3312.95)	0.15

^*a*^Summary statistic is absolute count (%) for categorical variables and median (IQR) for continuous data

^*b*^test statistic for categorical variables is the *χ* ^2^ test (6 df), and for continuous variables is the Kruskall-Wallis test

^C^ Glasgow coma score groups were defined as ‘normal’ if the score was 15/15, ‘mild impairment’ if the score was 13 or 14, ‘moderate impairment’ if the score was 11 or 12, and ‘severe impairment’ if the score was <11.

**Fig 1:**
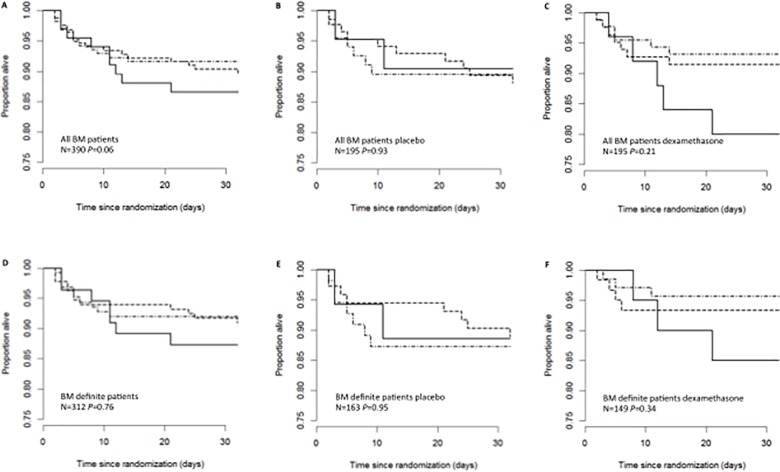
*LTA4H* genotypes, dexamethasone and survival in bacterial meningitis patients. Kaplan-Meier estimates of survival among BM patients stratified by their rs17525495 genotype in, (A) all BM patients (bacterially confirmed and probable), (B) all BM patients who received placebo, (C) all BM patients who received dexamethasone (D) definite BM patients (bacterially confirmed), (E) definite BM patients who received placebo and (F) definite BM patients who received dexamethasone. Solid line denotes TT, dash line denotes CT, and dot-dash line denotes CC genotype group. Comparisons of survival between genotypes were based on Cox regression models adjusted for the randomized treatment group

Thirty-nine patients died during the one month of follow-up. Survival curves stratified by rs17525495 genotype are displayed in Fig. 1 and corresponding Cox regression outputs are in S1 Table. No significant differences in survival between the three genotypes groups were found (all P>0.20). Moreover, there was no evidence that response to dexamethasone differed by genotype (interaction tests: P = 0.32 (all BM) and P = 0.41(definite BM)). There was also no evidence for an effect of genotype on the composite endpoint of death or severe neurological sequelae or bilateral severe deafness at 1 month. (S2 Table)

Here, we show that only TT homozygous individuals, with a predicted pro-inflammatory phenotype, are more susceptible to bacterial meningitis, but, unlike TBM, we found no evidence that *LTA4H* genotype influences response to treatment with dexamethasone. There may be a number of explanations for this observation, including differences in the pathophysiology of tuberculous and bacterial meningitis and differences in the duration of dexamethasone therapy given for each disease (4 days for bacterial meningitis versus 8 weeks for TBM). Also, adjunctive dexamethasone improves survival from TBM [13] but not bacterial meningitis [4], suggesting dexamethasone acts on different, and disease-specific, inflammatory pathways. Furthermore, a limitation of the study is that the observed number of deaths was low, thus power to detect differences are limited. Additionally, it is possible that the pathogenesis and mechanisms of bacterial meningitis caused by different pathogens (ie. *S*.*suis* and *S*.*pneumoniae*) could differ and affect our observed results. It is a further limitation of our study that we were unable to perform analysis by infecting organism due to small sample sizes and reduced power to detect associations. As *S*.*suis* only rarely causes bacterial meningitis in other settings our results should also be interpreted with caution in other countries.

Comparison of the clinical presentation of tuberculous and bacterial meningitis illustrates some of the key differences in pathophysiology. TBM presents with many days or even weeks of symptoms and the inflammation in the CSF is characterised by a moderate increase in white cells, the majority of which are mononuclear [13]. In contrast, pyogenic bacterial meningitis present acutely, with hours rather than days of symptoms, and with large numbers of neutrophils in both peripheral blood and CSF [15]. These clinical differences point to fundamental differences in the host response to infection with the respective pathogens. Macrophages are the cells primarily infected by *M*. *tuberculosis*, but they are also responsible for its control through the development of granuloma. In contrast, neutrophils are the key effector cells in pyogenic bacterial infections. Tobin *et al* showed that the *LTA4H* genotype influences macrophage response to mycobacterial infection [8]. Neutrophil recruitment may be influenced by *LTA4H* genotype by diverting eicosanoid synthesis to production of either the pro-inflammatory, neutrophil chemoattractant, LTB4, or the anti-inflammatory lipoxin A4. But in the model studied, these responses were driven by initial macrophage infection and there is no basis for believing a pyogenic bacterial infection would engage the same pathways. Lastly, it is possible that the failure to observe any effect of dexamethasone on survival from bacterial meningitis, or any interaction of its effect with *LTA4H* genotype, may be because it is only given for 4 days. This explanation seems less likely, given data from animal models have suggested early initial control of the inflammatory response by corticosteroids is sufficient to alter disease pathophysiology and improve outcome from bacterial meningitis [16].

*LTA4H* genotype is associated with susceptibility to bacterial meningitis in the Vietnamese. However we have no evidence to indicate *LTA4H* genotype is a critical determinant of bacterial meningitis outcome in the response to dexamethasone therapy.

## Supporting Information

S1 Table Association between rs17525495 genotype and 1-month mortality. (DOCX)

S2 Table Association between rs17525495 genotype and death or severe neurological sequelae or bilateral severe deafness at 1 month. (DOCX)
