# Supplementary material for: LTA4H Genotype Is Associated with Susceptibility to Bacterial Meningitis but Is Not a Critical Determinant of Outcome
Source: PLoS One. 2015 Mar 23;10(3):e0118789. doi: 10.1371/journal.pone.0118789 (PMC4370666; doi:10.1371/journal.pone.0118789)
Supplement: S1 Table — (DOCX) [file pone.0118789.s001.docx]

### S1 Table. Association between rs17525495 genotype and 1-month mortality

|  | **# deaths /# patients (%)** | **HR (95%CI) [compared to CC]** | **p-value** |
| --- | --- | --- | --- |
| All BM (n=390)*  CC (baseline)  CT  TT | 13/155 ( 8%)  17/168 (10%)  9/67 (13%) | -  1.20 (0.58-2.48)  1.57 (0.66-3.71) | 0.60 |
| All BM - Dexamethasone (n=195)  CC (baseline)  CT  TT | 6/88 ( 7%)  7/82 ( 9%)  5/25 (20%) | -  1.27 (0.43-3.77)  3.00 (0.92-9.83) | 0.21 |
| All BM - Placebo (n=195)  CC (baseline)  CT  TT | 7/67 (10%)  10/86 (12%)  4/42 (10%) | -  1.11 (0.42-2.92)  0.89 (0.26-3.05) | 0.93 |
| Definite BM (n=312)*  CC (baseline)  CT  TT | 10/124 ( 8%)  12/133 ( 9%)  7/ 55 (13%) | -  1.06 (0.45-2.45)  1.43 (0.54-3.78) | 0.76 |
| Definite BM – Dexamethasone (n=149)  CC (baseline)  CT  TT | 3/69 ( 4%)  4/60 ( 7%)  3/20 (15%) | -  1.57 (0.35-7.00)  3.44 (0.69-17.06) | 0.34 |
| Definite BM – Placebo (n=163)  CC (baseline)  CT  TT | 7/55 (13%)  8/73 (11%)  4/35 (11%) | -  0.85 (0.31-2.33)  0.88 (0.26-3.01) | 0.95 |

Results based on Cox regression; HR=hazard ratio.

p-values corresponds to overall likelihood ratio tests whether genotype plays a role.

* HR and p-values adjusted for treatment group; p-value for a treatment group-genotype interaction are p=0.32 (all BM), p=0.41(definite BM)
