# Supplementary material for: LTA4H Genotype Is Associated with Susceptibility to Bacterial Meningitis but Is Not a Critical Determinant of Outcome
Source: PLoS One. 2015 Mar 23;10(3):e0118789. doi: 10.1371/journal.pone.0118789 (PMC4370666; doi:10.1371/journal.pone.0118789)
Supplement: S2 Table — (DOCX) [file pone.0118789.s002.docx]

**S2 Table. Association between rs17525495 genotype and death or severe neurological sequelae or bilateral severe deafness at 1 month.**

|  | **# events / # patients (%)** | **OR (95%CI) [compared to CC]** | **p-value** |
| --- | --- | --- | --- |
| All BM (n=378)*  CC (baseline)  CT  TT | 31/150 (21%)  41/164 (25%)  15/ 64 (23%) | -  1.24 (0.73-2.12)  1.09 (0.53-2.19) | 0.73 |
| All BM - Dexamethasone (n=190)  CC (baseline)  CT  TT | 14/86 ( 16%)  17/80 (21%)  6/24 (25%) | -  1.39 (0.63-3.08)  1.71 (0.54-4.95) | 0.55 |
| All BM - Placebo (n=188)  CC (baseline)  CT  TT | 17/64 (27%)  24/84 (29%)  9/40 (23%) | -  1.11 (0.54-2.32)  0.80 (0.31-2.00) | 0.77 |
| Definite BM (n=307)*  CC (baseline)  CT  TT | 23/121 (19%)  33/131 (25%)  13/ 55 (24%) | -  1.38 (0.75-2.55)  1.22 (0.55-2.64) | 0.58 |
| Definite BM – Dexamethasone (n=146)  CC (baseline)  CT  TT | 10/67 (15%)  13/59 (22%)  4/20 (20%) | -  1.61 (0.65-4.10)  1.43 (0.35-4.92) | 0.58 |
| Definite BM – Placebo (n=161)  CC (baseline)  CT  TT | 13/54 (24%)  20/72 (28%)  9/35 (26%) | -  1.21(0.54-2.77)  1.09 (0.40-2.90) | 0.89 |

Results based on logistic regression; OR=hazard ratio. p-values corresponds to overall likelihood ratio tests whether genotype plays a role. 95% confidence intervals and p-values are based on likelihood ratio statistics.

* Outcome was missing in 12 subjects with BM and 5 subjects with definite BM. OR and p-values adjusted for treatment group; p-value for a treatment group-genotype interaction are p=0.59 (all BM), p=0.89 (definite BM)
